# Quality of life in a high-risk group of elderly primary care patients: characteristics and potential for improvement

**DOI:** 10.1007/s11136-024-03647-7

**Published:** 2024-05-13

**Authors:** Juliane Döhring, Martin Williamson, Christian Brettschneider, Thomas Fankhänel, Melanie Luppa, Alexander Pabst, Marina Weißenborn, Isabel Zöllinger, David Czock, Thomas Frese, Jochen Gensichen, Wolfgang Hoffmann, Hans-Helmut König, Jochen René Thyrian, Birgitt Wiese, Steffi Riedel-Heller, Hanna Kaduszkiewicz

**Affiliations:** 1 Institute of General Practice, University of Kiel, 24105 Kiel, Germany; 2 Institute of Social Medicine, Occupational Health and Public Health (ISAP), Medical Faculty, University of Leipzig, 04103 Leipzig, Germany; 3 Department of Health Economics and Health Services Research, University Medical Center Hamburg-Eppendorf, 20246 Hamburg, Germany; 4 Department of Clinical Pharmacology and Pharmacoepidemiology, University Hospital Heidelberg, 69120 Heidelberg, Germany; 5 Institute of General Practice/Family Medicine, University Hospital of LMU Munich, 80336 Munich, Germany; 6 Institute of General Practice and Family Medicine, Martin-Luther-University Halle-Wittenberg, 06112 Halle (Saale), Germany; 7 Work Group Medical Statistics and IT-Infrastructure, Hannover Medical School, Institute for General Practice, 30625 Hannover, Germany; 8 Institute for Community Medicine, University Medicine Greifswald, 17489 Greifswald, Germany; 9 Deutsches Zentrum für Neurodegenerative Erkrankungen (DZNE), 17489 Greifswald, Germany

**Keywords:** Quality of life, Prevention, Lifestyle, Late life, Risk factors, Primary care

## Abstract

**Purpose:**

Quality of Life (QoL) is associated with a bandwidth of lifestyle factors that can be subdivided into fixed and potentially modifiable ones. We know too little about the role of potentially modifiable factors in comparison to fixed ones. This study examines four aspects of QoL and its associations with 15 factors in a sample of elderly primary care patients with a high risk of dementia. The main objectives are (a) to determine the role of the factors in this particular group and (b) to assess the proportion of fixed and potentially modifiable factors.

**Method:**

A high-risk group of 1030 primary care patients aged between 60 and 77 years (52.1% females) were enrolled in “AgeWell.de,” a cluster-randomized, controlled trial. This paper refers to the baseline data. The multi-component intervention targets to decrease the risk of dementia by optimization of associated lifestyle factors. 8 fixed and 7 modifiable factors potentially influencing QoL served as predictors in multiple linear regressions.

**Results:**

The highest proportion of explained variance was found in psychological health and age-specific QoL. In comparison to health-related QoL and physical health, the modifiable predictors played a major role (corr. *R*^2^: 0.35/0.33 vs. 0.18), suggesting that they hold a greater potential for improving QoL.

**Conclusion:**

Social engagement, body weight, instrumental activities of daily living, and self-efficacy beliefs appeared as lifestyle factors eligible to be addressed in an intervention program for improving QoL.

**Trial registration:**

German Clinical Trials Register, reference number: DRKS00013555. Date of registration: 07.12.2017.

**Supplementary Information:**

The online version contains supplementary material available at 10.1007/s11136-024-03647-7.

## Introduction

According to the definition of the World Health Organization (WHO), Quality of life (QoL) is a multifactorial and broad concept reflecting an individual's perception of their position in life in the context of the culture and value systems in which they live and in relation to their goals, expectations, standards, and concerns [1]. Various approaches to a definition distinguish between the psychological and the physical aspect as the “core areas” constituting QoL [2]. In the 1980s the term “health-related QoL (HRQoL)” was coined in order to differentiate it as a general medical concept from the use in other professions [3]. In comparison to QoL, HRQoL focuses on health in a narrower sense, including the medical definition and the fundamental importance of independent physical, emotional, and social functioning [4], for example mobility, the ability to fend for oneself, daily activities, pain and physical complaints, and anxiety/depression [5]. Although hard to quantify, QoL is one of the most pursued target values which is implicitly proposed in health care research. Health-related interventions for elderly people often prioritize improvement of QoL over other health-related parameters, since elderly people often struggle with age-specific adversities such as impairment of sensory functions, dependence on other people, lower social participation, fear of dying, and others [6].

Identifying predictors of QoL is a subject of intensive research in several disciplines [7]. In the present study, we included 15 predictors of QoL of elderly primary care patients with an increased risk of dementia.

The patients participated in the AgeWell.de study, a cluster-randomized controlled intervention trial. The main objective of AgeWell.de was an improvement of lifestyle factors that are known to contribute to a development of dementia [8]. Hence, the design of the intervention required a distinction between lifestyle factors, that are fixed (such as age or sex) and those ones that are potentially modifiable and have the potential to be changed deliberately (such as physical or cognitive activity). A substantial proportion of lifestyle factors contributing to dementia are also associated with various aspects of QoL, which was a secondary outcome of AgeWell.de. Accordingly, the predictors of QoL presented here were components of a pre-existing set of data and have subsequently been selected under the prerequisite that associations with QoL have been reported in the literature. We identified 8 fixed and 7 potentially modifiable predictors fulfilling the aforementioned conditions. The fixed predictors can be assigned to 3 major areas:

### Sociodemographic characteristics

We included 5 sociodemographic characteristics in our analysis: besides sex and age three predictors related to the socioeconomic status (income, education, vocational qualification) were considered. Previous studies have shown that males tend to have a higher QoL than females [9] and that QoL in older age groups decreases slightly in general, especially in connection with multimorbidity [10]. The associations between socioeconomic status and QoL vary depending on the measured aspects, but in general poorer socioeconomic status is associated with poorer QoL. This association tends to be weaker in the older age groups [11].

### Cognitive performance

Cognitive complaints have robust associations with QoL [12]. As cognitive capacities decline (due to dementia or other clinical conditions) negative effects on QoL have been shown [13]. As two aspects of cognitive performance, we used a global measure of cognitive functioning (comprising language skills, abstraction capabilities, attention and others) and memory function.

### Instrumental activities of daily living

Instrumental activities are defined as a set of more complex skills that are needed in order to live independently [14], such as managing the medicines, shopping for groceries on one’s own or managing money, and paying bills. Previous studies consistently showed a decline of QoL depending on the extent of needed external help in daily life [15].

The 7 modifiable predictors can be assigned to the following 4 areas:

### Social integration

Social engagement in general is considered as one of the most important predictors of QoL [16]. In older age, social contact usually declines and it has been shown that the onset of social engagement markedly increases HROoL [17]. Besides social engagement, we considered the household type (living alone or together with relatives) as living alone is associated with lower QoL in elderly persons [18].

### Physical and somatic health-related factors

Many physical predictors have been investigated in the context of QoL [19]. One well-investigated correlate of the physical constitution is the body mass index (BMI), which is known to influence various aspects of well-being [20]. A further aspect refers to the physical activity as an extensively studied factor associated with QoL [21].

### Psychological and mental health-related factors

Depression is one of the most powerful predictors of QoL in elderly people [22] as well as self-efficacy beliefs, meaning the optimistic expectation of one´s own competency to master difficult situations successfully [23]. High self-efficacy beliefs contribute positively to QoL in many respects [24], elderly people with high self-efficacy beliefs, for example, tend to an improved compliance with self-care activities [25].

### Cognitive activity

In this context, cognitive activities are defined as common everyday activities demanding mental effort, for example solving crossword puzzles or reading books. Findings with respect to cognitive activity affecting QoL are mixed [26]. These inconsistencies in the literature result partly from confounders, the broad variety of concepts and defined periods of time (immediate effects of cognitive activity vs. effects of cognitive activity throughout the lifespan).

This list covers a bandwidth of commonly identified predictors of QoL and makes no claim to completeness from a purely academical point of view. Instead, we put an emphasis on the distinction between fixed and potentially modifiable predictors against a background of practical relevance. On that note the present study should make a contribution to the changing potential of lifestyle factors that are associated with QoL. There is a large body of literature concerning QoL in dementia patients [27] but QoL in elderly primary care patients with high risk of dementia is only marginally investigated in general. A systematic literature review on this issue can be found in [28].

The main objective of the present study is to analyze if the fixed and modifiable predictors provide information about QoL in elderly primary care patients with an increased risk of dementia. Accordingly, this objective is construed as a prediction model. The results will deliver implications for future intervention programs with a main focus on QoL improvement. The results may also be useful for basic research concerning QoL in the elderly as they encompass a high amount of differentiated data in a large sample (*N* = 1030) with high risk of dementia.

## Materials and methods

### The AgeWell.de study

Data for this analysis were derived from the AgeWell.de study. AgeWell.de is a multi-component, cluster-randomized, controlled intervention study, which targets to decrease the risk of dementia in a high-risk group of elderly primary care patients [29]. Due to the lack of effective dementia treatment options, AgeWell.de was designed as a prevention study in order to improve the individual risk factor profile of the participants. A wide range of potential risk factors for dementia has been identified in the last decades: some of them are fixed and cannot be modified, such as a genetic disposition. Others are potentially modifiable. Some of the latter were addressed in the intervention, which were in detail: counseling on nutrition and ways to increase physical activity, cognitive training, optimization of medication and the management of cardiovascular risk factors, counseling on improvement of social activity and intervening in case of loss, grief, and depressive symptoms. Several lifestyle factors associated with dementia are also known to contribute to QoL. The current paper is a secondary analysis of the AgeWell.de- baseline data referring to associations between lifestyle risk factors and QoL as a secondary outcome of the entire study. The primary outcome of the entire study is the preservation of cognitive function and delayed cognitive decline, respectively.

### Sample

The 1030 AgeWell.de-participants were recruited by 123 general practices (each corresponding to one cluster) in five German cities (Leipzig, Greifswald, Kiel as well as Halle are medium-sized cities and Munich is a large city) between 2018 and 2019. The participating general practitioners (who agreed to support the study after a written request to all possible eligible practices) identified possible participants among their patients based on the following criteria: Persons aged 60–77 with a CAIDE score ≥ 9. The CAIDE score (Cardiovascular Risk Factors, Aging, and Incidence of Dementia) is a validated tool to calculate late-life dementia risk based on midlife vascular risk factors [30]. Exclusion criteria were diagnosis of dementia, residence in a nursing home, poor German language skills, simultaneous participation in another intervention study, and severe mental or physical illness. With respect to the latter, the practitioners had to estimate individually, if the patient is potentially healthy enough to partake in the intervention. Patients, who fulfilled the criteria, were asked for study participation in a written form. Those patients, who showed interest to participate, were invited by the practitioners, received further information about the study, and gave written agreement. A total of 1176 persons initially consented to participate. 44 (3.7%) were not eligible (mainly because they did not meet the CAIDE criteria). 102 (8.7%) dropped out before the baseline assessment due to the occurrence of health problems, relocation, or withdrawal of consent. There was no difference between eligible and non-eligible participants with respect to age and sex, but the latter had significantly more years of education and a lower CAIDE score. More details about the recruitment procedures can be found in [31].

### Outcome measures: quality of life

Four QoL-outcome measures were selected in order to reflect (1) HRQoL, (2) physical health, and (3) psychological health as well as 4) age-specific QoL:

The first outcome measure represents a global scale of HRQoL. It was operationalized by the EuroQol-5 Dimension (EQ-5D), which is a generic questionnaire including the following five dimensions: “mobility,” “self-care,” “usual activities,” “pain/discomfort,” and “anxiety/depression.” The concept of health in EQ-5D also encompasses positive aspects (well-being) as well as negative aspects (illness) [4]. The EQ-5D data presented here were transformed into utilities according to the German value set [32]. The second outcome parameter is a quantitative measure of the self-perceived state of physical health, using the visual analog scale (EQ VAS, EuroQol Group, 1990), which ranges from 0 (worst imaginable health status) to 100 (best imaginable health status). Third, we used The World Health Organization Quality of Life Abbreviated Version (WHOQOL-BREF) as a patient reported outcome (PRO) questionnaire, quantifying the global health state, which refers to the aforementioned broad WHO definition and is independent from disabilities [5]. The WHOQOL-BREF assesses four domains of health, which are calculated and interpreted separately. The domains comprise physical and psychological health, social relationships, and environment. The answer format is a five-point Likert scale for every single item. The sum score for every domain has to be divided by the number of items per domain and subsequently multiplied by 4. All scores are multiplied by 4 to make them comparable with the scores derived from the WHOQOL-100. In this study, we paid special attention to the second domain as a measure of the self-perceived state of psychological health. The results for the other three domains are listed in an electronic supplement (Table 2). The fourth outcome measure takes into account the advanced age of our sample. The *World Health Organization Quality Of Life Instrument-Older Adults Module* (WHOQOL-OLD) was applied as a measure regarding age-specific domains of QoL. This questionnaire provides a global estimation of the following six relevant facets of QoL in higher age: sensory functions, independence, activities in the past, present and future, social participation, worries concerning death, and intimacy. As a global measurement of age-specific QoL, we calculated the total score based on the six individual facets. The results for the individual facets are also listed in the electronic supplement (tables 3 and 4).

### Predictor variables: fixed and modifiable predictors

Eight fixed predictors were included in the model. *Sociodemographics* comprise five predictors*:* age, sex, and three indices of the socioeconomic status (SES) according to [33]. The SES is a well-established analytical concept for epidemiologic research and health reporting in Germany and is based on information concerning education and vocational training as well as well as household net income. The SES indices are calculated as total scores including income, education, and vocational qualification. The *cognitive performance* was differentiated by cognitive function (Montreal Cognitive Assessment, Moca [34]) and memory function (Consortium to Establish a Registry for Alzheimer's Disease, CERAD: word list recall [35]). The *instrumental activities of daily living* were measured by the IADL-Skala [36].

In addition, 7 modifiable predictors, which also relate to QoL, were included. *Social integration* is represented by the predictors social engagement in general (Lubben social network scale, LSNS-6 [37]) and living in a single-person household*.* As *physical and somatic health-related factors,* we used the BMI and a self-constructed questionnaire for physical activity. The latter consists of 10 items (e.g., “how often do you ride the bike?”) and was scored by adding the answers on a five-point Likert scale (the entire questionnaire is available as supplementary information). *Psychological and mental health-related factors* refer to depression (Geriatric Depression Scale, GDS [38]) and self-efficacy (Skala zur allgemeinen Selbstwirksamkeitserwartung, SWE [39]). C*ognitive activity* was also measured by a self-constructed questionnaire, analogous to the questionnaire for physical activity. It consists of 12 items (e.g., “how often do you solve crossword puzzles?”) and is also available as supplementary information. Table 1 shows a detailed list of the outcome measures and predictors as well as according descriptive characteristics of the sample.

**Table 1:** Characteristics of the study population concerning outcome measures and predictors

Outcome measures	Scale	*N*	Measured values: range	Mean (SD)
Health-related QoL	EuroQol-5 Dimension (EQ 5-D)	1025	0.005–1.0	0.9 (0.1)
Psychological health	The World Health Organization Quality of Life Abbreviated Version (WHOQOL-BREF), 2nd Domain	987	5.33–20.00	15.7 (2.1)
Physical health	Visual analog scale (EQ VAS)	1026	9–100	76.4 (15.9)
Age-specific QoL	World Health Organization Quality of Life Instrument-Older Adults Module (WHOQOL-OLD), total score	1001	22.92–151.67	73.8 (12.4)

Modifiable predictors	Scale	*N*	Measured values: range	Mean (SD)/frequency (%)
Single-person household	Dichotomous	1030	Alone With others	297 (28.8%) alone 733 (71.2%) with others
Social engagement	Lubben social network scale (LSNS)	798	3–38	20.8 (6.6)
Body mass index	Body weight (kg)/body height (m^2^)	1014	19.6–55.1	31.0 (5.5)
Physical activity	Self-constructed questionnaire	1030	0–90	15.8 (10.3)
Depression	Geriatric depression scale (GDS)	1016	0–11	1.6 (2.0)
Self-efficacy	Skala zur allgemeinen Selbstwirksamkeitserwartung (SWE)	1023	5–90	24.6 (12.1)
Cognitive activity	Self-constructed questionnaire	1030	0–108	32.8 (13.6)

Fixed predictors	Scale	*N*	Measured values: range	Mean (SD)/frequency
Age	Years during baseline	1030	60–78	69.0 (4.9)
Sex	Dichotomous	1030	Female Male	537 (52.1) females 493 (47.9) males
Income	SES-index	930	1–7	4.2 (1.9)
Education	SES-index	1030	1–7	4.1 (1.4)
Vocational qualification	SES-index	1030	1–7	3.2 (1.0)
Cognitive function	Montreal Cognitive Assessment (Moca)	1027	9–30	24.3 (3.1)
Memory function	Consortium to Establish a Registry for Alzheimer's Disease (CERAD, Word list recall)	1002	0–10	5.7 (2.3)
Instrumental activities of daily living	Lawton and Brody instrumental activities of daily living (IADL) scale	1024	0–42.75	1.24 (3.73)

Reliabilities for the outcome measures: EQ-5D: ICC: 0.7 [53], WHOQOL-BREF, 2nd domain: test–retest reliability: 0.72 [54], EQ VAS: ICC: 0.65 [55], WHOQOL-OLD: ICC: 0.9 [56]

### Statistical analyses

Descriptive statistical analyses (means and frequencies) concerning the baseline data of the AgeWell.de cohort were conducted for the four QoL-outcome measures (primary endpoints) and the 15 predictor variables.

Four multiple linear regressions were performed in order to estimate the predictive values of all 15 considered predictors (i.e., goodness-of-fit in total) and the sets of predictors (fixed and modifiable) on the QoL-outcome measures. Bivariate relationships between the predictors and outcome measures are listed in the electronic supplement (Table 5).

Statistically significant coefficients of determination (corrected *R*^2^), unstandardized regression coefficients b, standardized regression coefficients β, and p values are shown. All statistical analyses were performed using IBM SPSS software V22.0 (SPSS Inc., Chicago, IL, USA).

## Results

### Descriptive statistics

1030 AgeWell.de-participants were included in the analyses. 52.1% of the participants were females. At the date of baseline measurement (to which this analysis refers), the participants were 69.0 (SD 4.9) years old in average.

### Regression analyses: predictive values of the entire models, modifiable, and fixed predictors

All *R*^2^ were highly significant (*p* < 0.001) without exception.

The coefficients of determination (*R*^2^) for the overall model regarding HRQoL (EQ 5-D) were indicative for a moderate goodness-of-fit according to Cohen [44]. This outcome measure was explained by the modifiable and the fixed predictors to similar proportions (Table 2).

**Table 2:** Explained variance of the four QoL-outcome measures by means of the modifiable and fixed predictor groups

Outcome measures	*N*	All predictors corr. *R*^2^	Modifiable predictors corr. *R*^2^	Fixed predictors corr. *R*^2^
Health-related QoL (EQ 5-D)	1025	0.20	0.18	0.16
Psychological health (WHOQOL-BREF), 2nd domain	987	0.41	0.35	0.13
Physical health (EQ VAS)	1026	0.21	0.18	0.11
Age-specific QoL (WHOQOL-OLD)	1001	0.34	0.33	0.09

The goodness-of-fit (model accuracy) is highly significant (*p* value less than 0.01) in all combinations of outcome measures and sets of predictors

*EQ 5-D* EuroQol-5 Dimension, *WHOQOL-BREF* The World Health Organization Quality of Life Abbreviated Version, *EQ VAS* Physical health (visual analog scale), *WHOQOL-OLD* World Health Organization Quality of Life Instrument-Older Adults Module

The highest proportion of explained variance was found in the domains of psychological health (WHOQOL-BREF), mainly in connection with modifiable predictors. A similar pattern also applies to age-specific QoL (WHOQOL-OLD). The goodness-of-fit in psychological health as well as age-specific QoL has to be classified as high.

The physical health (EQ VAS) could be explained to a moderate extent in general, and to a lower degree by fixed predictors compared to modifable ones (Tables 3 and 4).

**Table 3:** Fixed predictors of Quality of life in elderly primary care patients with high risk of dementia

Fixed predictor	Health-related QOL	Psychological health	Physical health	Age-specific QOL
Age	0.002/0.062 (0.00, 0.004)	− 0.001/− 0.005 (− 0.02, 0.02)	0.011/0.002 (− 0.21, 0.23)	− 0.051/− 0.021 (− 0.21, 0.11)
Male sex	0.022*/0.080 (0.00, 0.04)	0.568**/0.129 (0.29, 0.85)	0.526/0.015 (− 1.78, 2.82)	− 0.553/− 0.023 (− 2.19, 1.08)
SES income	0.004/0.063 (0.00, 0.01)	0.018/0.019 (− 0.06, 0.10)	0.505/0.065 (− 0.14, 1.15)	0.346/0.055 (− 0.11, 0.81)
SES education	0.009*/0.094 (0.00, 0.02)	0.104/0.068 (0.00, 0.21)	0.380/0.032 (− 0.51, 1.27)	0.233/0.027 (− 0.40, 0.86)
SES vocational qualification	− 0.002/− 0.007 (− 0.01, 0.01)	− 0.283**/− 0.128 (− 0.44, 0.13)	− 1.172/− 0.070 (2.47, 0.13)	− 0.756/− 0.061 (− 1.68, 0.17)
Cognitive function	− 0.002/− 0.059 (− 0.01, 0.00)	0.035/0.043 (− 0.01, 0.08)	− 0.305/− 0.063 (− 0.70, 0.09)	− 0.082/− 0.022 (− 0.36, 0.20)
Memory function	0.003/0.050 (0.00, 0.01)	− 0.018/− 0.017 (− 0.08, 0.04)	0.430/0.067 (− 0.05, 0.91)	− 0.039/− 0.006 (− 0.38, 0.30)
IADL	− 0.004*/− 0.093 (− 0.01, 0.00)	− 0.063*/− 0.093 (− 0.11, 0.02)	− 0.498*/− 0.102 (− 0.86, 0.14)	− 0.137/− 0.036 (0.39, 0.12)

*SES* socioeconomic status

The standardised coefficient *β* is presented right to the unstandardized coefficient b. Confidence intervals are reported in parentheses

*,** Indicates significance at the 95% and the 99% level, respectively

**Table 4:** Modifiable predictors of quality of life in elderly primary care patients with high risk of dementia

Modifiable predictor	Health-related QOL	Psychological health	Physical health	Age-specific QOL
Single-person household	− 0.005/− 0.017 (− 0.03, 0.02)	− 0.189/−0.039 (− 0.48, 0.11)	− 2.210/− 0.064 (− 4.66, 0.24)	− 2.003*/− 0.074 (− 3.74, 0.26)
Social engagement	0.000/0.013 (− 0.001, 0.002)	0.015/0.048 (− 0.01, 0.04)	0.079/0.036 (− 0.10, 0.26)	0.220**/0.120 (0.09, 0.35)
Body mass index	− 0.003**/− 0.119 (− 0.005, 0.001)	− 0.034*/− 0.090 (− 0.06, 0.01)	− 0.509**/− 0.183 (− 0.71, 0.31)	− 0.159*/− 0.073 (− 0.30, 0.02)
Physical activity	0.001/0.067 (0.00, 0.002)	0.004/0.019 (− 0.01, 0.02)	0.090/0.056 (− 0.03, 0.21)	− 0.020/− 0.016 (− 0.11, 0.07)
Depression	− 0.022**/− 0.355 (− 0.03, 0.02)	− 0.564**/− 0.565 (− 0.64, 0.49)	− 2.41**/− 0.351 (− 3.00, 1.82)	− 2.85**/− 0.491 (− 3.27, 2.43)
Self-efficacy	0.006/− 0.003 (− 0.001, 0.001)	0.025**/0.121 (0.01, 0.04)	0.016/0.002 (− 0.08, 0.11)	0.140**/0.125 (0.07, 0.21)
Cognitive activity	− 0.001/− 0.057 (− 0.002, 0.00)	− 0.003/− 0.006 (− 0.01, 0.01)	− 0.032/− 0.017 (− 0.13, 0.07)	0.007/0.011 (− 0.06, 0.08)

The standardized coefficient *β* is presented right to the unstandardized coefficient b. Confidence intervals are reported in parentheses

*,**Indicate significance at the 95% and the 99% level, respectively

### Regression analyses: influence of the individual fixed predictors

The ability to manage the IADL was significantly associated with the health-related QoL as well as psychological and physical health. Male sex was a significant predictor for a higher health-related QoL and better psychological health. The socioeconomic status played a lesser role, merely vocational qualification was significantly associated with psychological health, as well as higher education with health-related QoL. Income did not relate to the considered QoL instruments and domains. This also applies to age and cognitive function, as well as memory function as measured in standardized tests.

### Regression analyses: influence of the individual modifiable predictors

Body mass index and depression had a significant predictive value in all considered QoL-outcome measures. High self-efficacy beliefs were significant predictors with respect to psychological health and age-specific QoL.

Additionally, age-specific QoL was also positively associated with higher social engagement and living together with other people. There was no noteworthy relationship between physical and cognitive activity in all considered QoL instruments or domains, respectively.

## Discussion

The main objective of the present study was to analyze if the determinants of QoL known from the literature can also be found in elderly primary care patients with an increased risk of dementia. Based on these results, implications for future intervention programs with a focus on QoL improvement are discussed.

### Fixed predictors of Quality of Life

We found significant associations between IADL and health-related QoL as well as psychological and physical health. This is in line with previous studies that consistently showed a decline of QoL dependent on the extent of needed external help in daily life [15]. IADL are related to physical and mental health in elderly people [45]. Based on the important role of IADL in QoL, one must consider whether IADLs are really fixed predictors. For example, improvements in IADL (and thus in the QoL) could be achieved through a critical evaluation of the medical treatment and, if necessary, a change in therapy [46]. Targeted training of IADL in case of limitations due to chronic diseases would also be conceivable in order to improve QoL. This is especially true for patients with cognitive impairment. Here, studies have shown that consistent IADL/ADL training can slow down the loss of these skills [47]. As expected, male sex was a significant predictor in general health-related QoL and psychological health. This finding is in line with the literature [48]. In our study population, income was not associated with the QoL domains. A possible explanation may be the selection of the QoL-outcome measurements that focused on aspects of well-being rather than evaluation of life [49]. Education and vocational qualification each contributed to one instrument/domain of QoL. In the literature, the effects of education on QoL are described as multidimensional and often reciprocal in nature [50]. The quality of life schema as a heuristic framework according to [51] includes educational effects in the following broad life domains: achievement in life, material and emotional well-being, physical health, community, intimate relationships, and personal safety/ future security.

Interestingly, age was not significantly related to the QoL domains, which may be due to the relatively small age-range in the study (60 to 78 years). Another factor contributing to this finding may be a result of the exclusion criteria leading to a sample without severely diseased persons. As pointed out in a narrative review of selected literature [52], with all other influences controlled, aging per se does not influence quality of life negatively.

Cognitive and memory function had also no noteworthy predictive value for QoL in our sample. We assume that this may be a result of ceiling effects.

### Modifiable predictors of Quality of Life

The modifiable predictors contributed considerably higher to the explained variance than the fixed predictors in the QoL domains, especially regarding psychological health and age-specific QoL.

One of the most remarkable results in this context is the predictive role of the BMI for QoL throughout all four instruments and domains applied. A high BMI affects QoL in various aspects: lower mobility, higher risk of cardiovascular diseases, and diabetes mellitus, as well as arthrosis, protracted courses, and complications of many diseases, lower self-esteem, and others. In turn, several mental illnesses may precipitate and perpetuate obesity [53]. The association between obesity and QoL is thus bidirectional and complex. It further stands to reason that elderly persons in particular suffer from the effects of obesity, because compensation opportunities become fewer in old age [54].

Depression, measured by the GDS, also contributed to the variance in all considered QoL instruments and domains. This association has been described in a broad range of literature and was found to be stable over time regardless of the assessed instruments for QoL [55]. It cannot be ruled out that conceptual overlaps between depression and QoL may play a role in this context. However, besides depression, other factors are also associated with the age-specific QoL domains, such as living alone or with others, social engagement, and self-efficacy. To improve QoL in old age, interventions focusing on social integration and BMI seem appropriate. This is a positive message of this study, as these factors can indeed be influenced. Whether it is possible to improve QoL through interventions at this age should be investigated in appropriate studies.

With respect to the modifiable factors, a surprising result was the fact that neither physical, nor cognitive activity significantly predicts QoL in our data, although the questionnaires covered a broad range of daily activities. This outcome may be a result of the short period of time to which the questions referred: the participants were asked for cognitive and physical activity within the last four weeks. Further analysis indicated that the predictors “BMI” and “physical activity” co-varied: excluding BMI from the regression model results in significant variance contribution of physical activity in the physical health domain as well as in the health-related QoL (p value = 0.02 in both domains). For a more detailed overview about physical activity determinants in this sample, see Cardona et al. [56].

### Quality of Life instruments and domains

All of the four considered QoL instruments and domains were explained by the multiple-regression models to a moderate to high goodness-of-fit. The predictive value of the models was particularly high in two domains: psychological health and age-specific QoL. Again, the substantial proportion was allocated to the modifiable predictors.

The physical health domain as well as the health-related QoL showed only a moderate goodness-of-fit. A reason may be that “physical health” in this context was a global estimation of self-perceived health, which was explicitly restricted to bodily aspects, according to the EQ VAS. The visual analog scale as a single item represents a rather limited concept in comparison to the other outcomes. It might be reasonably assumed that physical health (in comparison with psychological health and age-specific QoL) is determined rather by individual and external factors (such as genetic dispositions or injuries). This may also partly apply to the health-related QoL, measured by EQ 5D, which is composed of physical and mental parts.

## Limitations

Limitations of our data relate to the selection of predictors: improving QoL was a secondary objective in the AgeWell.de study. Accordingly, the set of predictors was restricted to factors potentially influencing the primary objective, i.e., the development of dementia. Taking more QoL-specific predictors into account might have led to a higher goodness-of-fit of the models. Furthermore, the aforementioned prioritization of the predictor selection entails a possible content overlap between some predictors and outcome measurements (e.g., depression and psychological health), which may lead to an overestimation of the actual empirical associations. Another limitation at this point of time is the cross-sectional approach. Therefore, it is not yet possible to draw conclusions about the causal links between predictors and outcomes as well as the relationships between predictors among each other. According to this, as a prediction model, our data do not provide information about how to increase QoL. The follow-up data of the AgeWell.de study will generate new evidence on causal links and interactions.

## Conclusions

This study provides important information on factors for optimization of QoL in elderly primary care patients with an increased risk of dementia. One of the most important predictors for age-specific QoL is social engagement in advanced age. Secondly, body weight seems to be another desirable key factor for well-being. Thirdly training and improvement of IADL seem desirable not only for themselves, but also with potential to improve QoL. Fourthly, a “psychological adjusting screw” refers to a shift from an external to an internal locus of control resulting in the belief, that one´s own action has (positive) consequences (self-efficacy beliefs). In sum, the data showed various associations between modifiable predictors, pointing on a high and realistic potential to improve QoL by concrete modifications on the behavioral, physical, and psychological level as stated above. Testing interventions with the aim to improve QoL in elderly patients with and without an increased risk of dementia should be a next step in research.

### Supplementary Information

Below is the link to the electronic supplementary material.

Supplementary file1 (DOCX 22 kb)

Supplementary file2 (DOCX 15 kb)
