# Supplementary material for: Quality of life in a high-risk group of elderly primary care patients: characteristics and potential for improvement
Source: Qual Life Res. 2024 May 13;33(7):1841–51. doi: 10.1007/s11136-024-03647-7 (PMC11176227; doi:10.1007/s11136-024-03647-7)
Supplement: Supplementary file 1 — Supplementary file1 (DOCX 22 kb) [file 11136_2024_3647_MOESM1_ESM.docx]

*Table 1. Fixed predictors of the other three WHOQOL-BREF domains*

| **fixed**  **predictor** | **b**  **physical health**  **(domain 1)** | **b**  **social relationships**  **(domain 3)** | **b**  **environmental health**  **(domain 4)** |
| --- | --- | --- | --- |
| **Age** | ,025 | **,035*** | **,037**** |
| **Male sex** | -,172 | **,417*** | -,165 |
| **SES income** | **,219**** | **,122*** | **,336**** |
| **SES education** | **,152*** | -,056 | ,030 |
| **SES vocational qualification** | ,031 | -,067 | ,097 |
| **Cognitive function** | ,012 | ,008 | ,037 |
| **Memory function** | ,009 | ,070 | ,025 |
| **Instrumental activities of daily living (IADL)** | **-,217**** | **-,106**** | **-,093**** |

SES: Socioeconomic status, * b = unstandardised regression coefficient b, *p-value ≤ .05, **p-value < 0.001

*Table 2. Modifiable predictors of the other three WHOQOL-BREF domains*

| **Modifiable**  **predictor** | **b**  **physical health**  **(domain 1)** | **b**  **social relationships**  **(domain 3)** | **b**  **environmental health**  **(domain 4)** |
| --- | --- | --- | --- |
| **Single-person household** | -,149 | ,047 | **,413*** |
| **Social engagement** | ,006 | **,059**** | **,056**** |
| **Body mass index** | **-,103**** | -,007 | **-,030*** |
| **Physical activity** | ,016 | -,004 | -,004 |
| **Depression** | **-,570**** | **-,488**** | **-,356**** |
| **Self-efficacy** | ,015 | ,007 | ,006 |
| **Cognitive activity** | ,002 | -,010 | **,015**** |

*b = unstandardised regression coefficient b, *p-value ≤ .05, **p-value < 0.001

*Table 3. Fixed predictors of the WHOQOL-OLD scores*

| **fixed**  **predictor** | **b**  **sensability** | **b**  **autonomy** | **b**  **past** | **b**  **social participation** | **b**  **death** | **b**  **intimacy** |
| --- | --- | --- | --- | --- | --- | --- |
| **Age** | **-,272*** | ,029 | ,126 | ,107 | ,291 | ,196 |
| **Male sex** | **3,434**** | **4,274**** | ,246 | -,072 | **-4,835**** | ,089 |
| **SES income** | **1,433**** | **,894**** | **1,212**** | **,951**** | **1,529**** | **1,311**** |
| **SES education** | ,365 | ,096 | ,072 | ,697 | -,142 | -,451 |
| **SES vocational qualification** | -,212 | ,793 | ,288 | ,257 | -,593 | ,225 |
| **Cognitive function** | **,440*** | ,291 | -,130 | -,084 | -,528 | ,138 |
| **Memory function** | ,119 | ,088 | ,154 | ,122 | ,198 | ,171 |
| **Instrumental activities of daily living (IADL)** | **-,894**** | **-1,037**** | **-,619**** | **-,716**** | **-,625**** | -,262 |

SES: Socioeconomic status, * b = unstandardised regression coefficient b, *p-value ≤ .05, **p-value < 0.001

*Table 4. Modifiable predictors of the WHOQOL-OLD scores*

| **Modifiable**  **predictor** | **b**  **sensability** | **b**  **autonomy** | **b**  **past** | **b**  **social participation** | **b**  **death** | **b**  **intimacy** |
| --- | --- | --- | --- | --- | --- | --- |
| **Single-person household** | -1,977 | ,594 | ,969 | ,902 | 3,153 | 1,230 |
| **Social engagement** | ,126 | **,246*** | **,235*** | **,205*** | **,323*** | **,303*** |
| **Body mass index** | -,071 | -,071 | **-,231*** | **-,410**** | ,158 | -,284 |
| **Physical activity** | -,029 | ,024 | ,031 | ,095 | -,073 | -,062 |
| **Depression** | **-2,695**** | **-2,988**** | **-3,419**** | **-3,418**** | **-3,406**** | **-2,389**** |
| **Self-efficacy** | ,004 | -,019 | ,057 | **,098*** | **,220**** | -,023 |
| **Cognitive activity** | **,135*** | ,060 | ,001 | -,007 | **-,153*** | ,031 |

*b = unstandardised regression coefficient b, *p-value ≤ .05, **p-value < 0.001

*Table 5. Bivariate relationships between the outcomes and fixed predictors*

| **fixed**  **predictor** | **Health-related QoL** | **Psychological health** | **Physical health** | **Age-specific QoL** |
| --- | --- | --- | --- | --- |
| **Age** | ,048  ,123 | ,014  ,671 | ,016  ,599 | ,002  ,950 |
| **Male sex** | **,128^**^**  **,000** | **,134^**^**  **,000** | ,041  ,195 | ,002  ,941 |
| **SES income** | **,194^**^**  **,000** | **,189^**^**  **,000** | **,183^**^**  **,000** | **,233^**^**  **,000** |
| **SES education** | **,156^**^**  **,000** | **,126^**^**  **,000** | **,111^**^**  **,000** | **,109^**^**  **,001** |
| **SES vocational qualification** | **,177^**^**  **,000** | **,098^**^**  **,002** | **,127^**^**  **,000** | **,130^**^**  **,000** |
| **Cognitive function** | **,061^*^**  **,049** | **,109^**^**  **,001** | **,092^**^**  **,003** | **,111^**^**  **,000** |
| **Memory function** | ,059  ,063 | ,042  ,190 | **,092^**^**  **,004** | **,084^**^**  **,009** |
| **Instrumental activities of daily living (IADL)** | **-,324^**^**  **,000** | **-,288^**^**  **,000** | **-,302^**^**  **,000** | **-,222^**^**  **,000** |

Pearson correlation coefficients (r) and significance level (*p-value ≤ .05, **p-value < 0.001)

*Table 6. Bivariate relationships between the outcomes and modifiable predictors*

| **Modifiable**  **predictor** | **health-related QoL** | **Psychological health** | **Physical health** | **age-specific QoL** |
| --- | --- | --- | --- | --- |
| **Single-person household** | ,003  ,932 | -,031  ,416 | -,020  ,588 | -,030  ,421 |
| **Social engagement** | **,156^**^**  **,000** | **,227^**^**  **,000** | **,179^**^**  **,000** | **,304^**^**  **,000** |
| **Body mass index** | **-,184^**^**  **,000** | **-,159^**^**  **,000** | **-,230^**^**  **,000** | **-,129^**^**  **,000** |
| **Physical activity** | ,060  ,056 | **,099^**^**  **,002** | **,112^**^**  **,000** | **,076^*^**  **,016** |
| **Depression** | **-,409^**^**  **,000** | **-,580^**^**  **,000** | **-,386^**^**  **,000** | **-,535^**^**  **,000** |
| **Self-efficacy** | ,052  ,095 | **,130^**^**  **,000** | ,029  ,355 | **,110^**^**  **,000** |
| **Cognitive activity** | ,038  ,225 | **,104^**^**  **,001** | **,067^*^**  **,031** | ,**131^**^**  **,000** |

Pearson correlation coefficients (r) and significance level (*p-value ≤ .05, **p-value < 0.001)
