# Supplementary material for: Quality of life in a high-risk group of elderly primary care patients: characteristics and potential for improvement
Source: Qual Life Res. 2024 May 13;33(7):1841–51. doi: 10.1007/s11136-024-03647-7 (PMC11176227; doi:10.1007/s11136-024-03647-7)
Supplement: Supplementary file 2 — Supplementary file2 (DOCX 15 kb) [file 11136_2024_3647_MOESM2_ESM.docx]

# Supplementary Information: physical activity and cognitive activity

*This questionnaire refers to the physical activity and cognitive activity over the last 4 weeks, unless there were strong reasons why the usual circumstances were changed, e.g. a hospital stay. In this case „the last 4 weeks“ refers to the normal situation, which will subsequently be reached again.*

| „In the next minutes I would like to ask you some questions about your activities over the last four weeks.“ | | | | | |
| --- | --- | --- | --- | --- | --- |
| How often… | **Every day** | **Several times per week** | **Once per week** | **Less than once per week** | **never** |
| …do you ride the bike? | □ | □ | □ | □ | □ |
| …do you hike or take a walk (more than 10 minutes walking time)? | □ | □ | □ | □ | □ |
| …do you go swimming? | □ | □ | □ | □ | □ |
| …do you do gymnastics (at least 10 minutes at a time)? | □ | □ | □ | □ | □ |
| …do you do fitness or power training? | □ | □ | □ | □ | □ |
| …do you do other sports, for example soccer, handball, basketball, volleyball, badminton, rowing, martial art etc.? | □ | □ | □ | □ | □ |
| …do you use a pedometer? | □ | □ | □ | □ | □ |
| …do you work in the house and the garden? | □ | □ | □ | □ | □ |
| …do you take care of other people, for example children, grandchildren, relatives, friends, sick spouse? | □ | □ | □ | □ | □ |
| …do you do something different, for example bowling, dancing, home trainer, low intensity running, golf? | □ | □ | □ | □ | □ |
| …do you solve crossword puzzles? | □ | □ | □ | □ | □ |
| …do you do memory training, memory exercises or mental exercises? | □ | □ | □ | □ | □ |
| …do you do card games or board games, parlour games or single-user games, chess? | □ | □ | □ | □ | □ |
| …have you been socially committed (support for needy persons, tutoring, volunteer work, in church, in a retirement home, political party, association?) | □ | □ | □ | □ | □ |
| …do you specifically learn something new? (e.g. learning a new sport, lessons in dancing, cooking, language courses, etc.) | □ | □ | □ | □ | □ |
| …do you read (books, journals, recipes)? | □ | □ | □ | □ | □ |
| …do you write (poems, stories or letters (including e-mails, painting)? | □ | □ | □ | □ | □ |
| …do you make music (playing instruments, singing in a choir or in community with others? | □ | □ | □ | □ | □ |
| …do you make/receive phone calls? | □ | □ | □ | □ | □ |
| Do you use… | | | | | |
| …a mobile phone? | □ | □ | □ | □ | □ |
| …a computer? | □ | □ | □ | □ | □ |
| …other technical devices, for example video player/ DVD player? | □ | □ | □ | □ | □ |

**Scoring (sum score):** Every day (4), several times per week (3), once per week (2), less than once per week (1), never (0)
